# A Case of Cutaneous Tuberculosis Presenting As Lupus Vulgaris in an Immunocompetent Indian Child During the Pandemic of COVID-19

**DOI:** 10.7759/cureus.27996

**Published:** 2022-08-14

**Authors:** Sankalp Yadav

**Affiliations:** 1 Medicine, Shri Madan Lal Khurana Chest Clinic, New Delhi, IND

**Keywords:** carcinoma, lupus vulgaris, cutaneous tb, tuberculosis, covid-19

## Abstract

Cutaneous tuberculosis is a rare finding in clinical settings. However, cases of this type of TB are commonly reported from the high burden countries. The diagnosis of cutaneous tuberculosis is difficult, especially due to the paucibacillary nature of this disease and its similarities with other cutaneous infections. Also, there is low sensitivity and specificity in almost all diagnostic tests. Therefore, it requires a detailed history, with a rigorous clinical workup to establish the diagnosis. The diagnosis becomes even more challenging when the situation is grave due to an ongoing pandemic. During the current pandemic of COVID-19, there was lesser attention paid to other diseases. Most of the outpatient departments like dermatology were shut and human resources were utilized for COVID-19 care. The author herein presents a case of cutaneous tuberculosis presenting as lupus vulgaris in an Indian child diagnosed during the third wave of the pandemic of COVID-19.

## Introduction

Tuberculosis (TB) is an age-old infectious disease caused by *Mycobacterium tuberculosis *and is a major public health issue [1]. The most common form is pulmonary TB but extrapulmonary TB (EPTB) is also reported in large numbers [2]. Extrapulmonary manifestations of TB could be cutaneous, pleura, abdomen, bone and joints, liver, spleen, lymph nodes, meninges, etc. [2]. In general, EPTB is seen in 8.4-13.7% of all cases of TB [3]. Cutaneous TB is comparatively a rare presentation constituting around 1-1.5% of the total EPTB presentations [4]. *M. tuberculosis, M. bovis*, and the attenuated bacille Calmette-Guerin organism can result in all forms of cutaneous TB [5]. Cutaneous TB could have different clinical manifestations like scrofuloderma, TB verrucosa cutis, orificial TB, tuberculous gumma, lupus vulgaris, tuberculous chancre, and acute cutaneous miliary [6]. Off all these lupus vulgaris and cervical scrofuloderma are the commonest [6].

The diagnosis of cutaneous TB is challenging [6,7]. It is based on absolute or relative criteria [7]. An absolute criterion involves identifying *M. tuberculosis* from a positive result on polymerase chain reaction (PCR), tissue culture, or inoculation on guinea pigs [6,7]. However, due to the paucibacillary nature of cutaneous TB, it is a formidable task [8]. Therefore, a relative criterion is also available that includes a detailed history and assessment of lesions, finding of an acid-fast bacillus (AFB) on lesions, active TB found on other organs, finding of a tuberculous granuloma on histopathological examination, positive tuberculin test, and responsiveness toward anti-TB medications [7].

Lupus vulgaris is a common form of cutaneous TB seen in individuals sensitized to *M. tuberculosis* [7]. There are five major clinical variations of lupus vulgaris, i.e., hypertrophic or vegetation, plaque, tumor-like, papular or nodular, and ulcerative types [9]. Other clinical forms include atrophic and mutilating types [5,9]. The author herein presents, an interesting case of lupus vulgaris in an immunocompetent Indian child. The case is important as the diagnosis was made during the time of the third wave of COVID-19 in India and it required a great commitment with judicious use of lab resources since the dermatology OPD were either shut down or were working with limited capacities. And the fear of COVID-19 in the general public along with local lockdowns had resulted in delayed presentations of such cases. Moreover, the diagnosis and management of paucibacillary TB with oversaturated healthcare systems was a noteworthy clinical outcome.

## Case presentation

A nine-year-old Indian child born to a low-income family came to the outpatient department with complaints of a skin lesion on the medial aspect of her left hand for one and a half months. She also complained of loss of appetite for one month. Her parents were daily-wage workers who left for their village due to the loss of jobs during the lockdowns in New Delhi. The child reported late as the third wave of COVID-19 was going on and thus parents did not consult the health center.

The patient was well six weeks ago when she developed a small about 2 mm x 1 mm pustule on the medial aspect of her left hand. The lesion grew gradually and covered nearly the complete surface of the medial aspect of the left hand. It was painless with no discharge.

Detailed history revealed that she had fever two months back for which over-the-counter antipyretic (paracetamol) was taken. Fever was evening rise in pattern with no rigors or chills and subsided after the antipyretic and there was no fever after five such episodes. There was no remarkable medical or surgical history. Also, there was no history of skin diseases, trauma, TB, or COVID-19 to her. However, there was a history of sputum-positive pulmonary TB in her brother who was treated three years back and was declared cured. She was not vaccinated against COVID-19.

General examination revealed a lean girl with vitals: pulse - 80/minute; blood pressure - 110/80 mmHg, respiratory rate - 18/minute, temperature - 98.6 degrees Fahrenheit, weight - 30 kg, and SpO_2 _- 99% on room air. There was no clubbing, icterus, pallor, lymphadenopathy, cyanosis, edema, or koilonychia.

**Figure 1:**
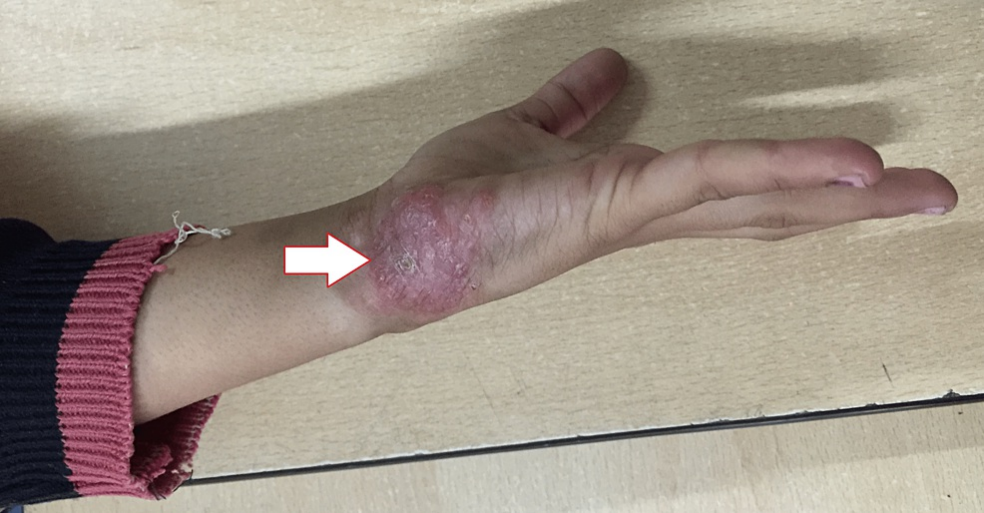
Well-marginated, reddish pink plaque

Systemic examination was unremarkable. Dermatological examination revealed a well-marginated, reddish pink plaque of 8 × 7 cm in size, with a rough surface, irregular edges, and mild scaling (Figure 1). Brownish-yellow or “apple-jelly” color on blanching by diascopic pressure was observed on the lesions. A dermatoscopic examination revealed yellow to orange areas on the lesions intersected by fine horizontal telangiectasia. These dermatoscopic findings backed by a family history of TB led to the consideration of granulomatous skin diseases. A skin biopsy was performed, which suggested thickened stratum corneum, irregular elongation of rete ridges, focal suprapapillary thinning, spongiosis, and lymphocytic exocytosis. The upper dermis showed dense lymphohistiocytic infiltrate, and epitheloid cell granulomas with Langhans giant cells. The deeper dermis also showed confluent epitheloid cell granulomas with occasional central necrosis. Stains for AFB were negative and findings were consistent with lupus vulgaris. Periodic acid-Shiff (PAS), and Erlich-Ziehl-Neelsen (EZN) staining could not detect any specific infectious agent.

**Figure 2:**
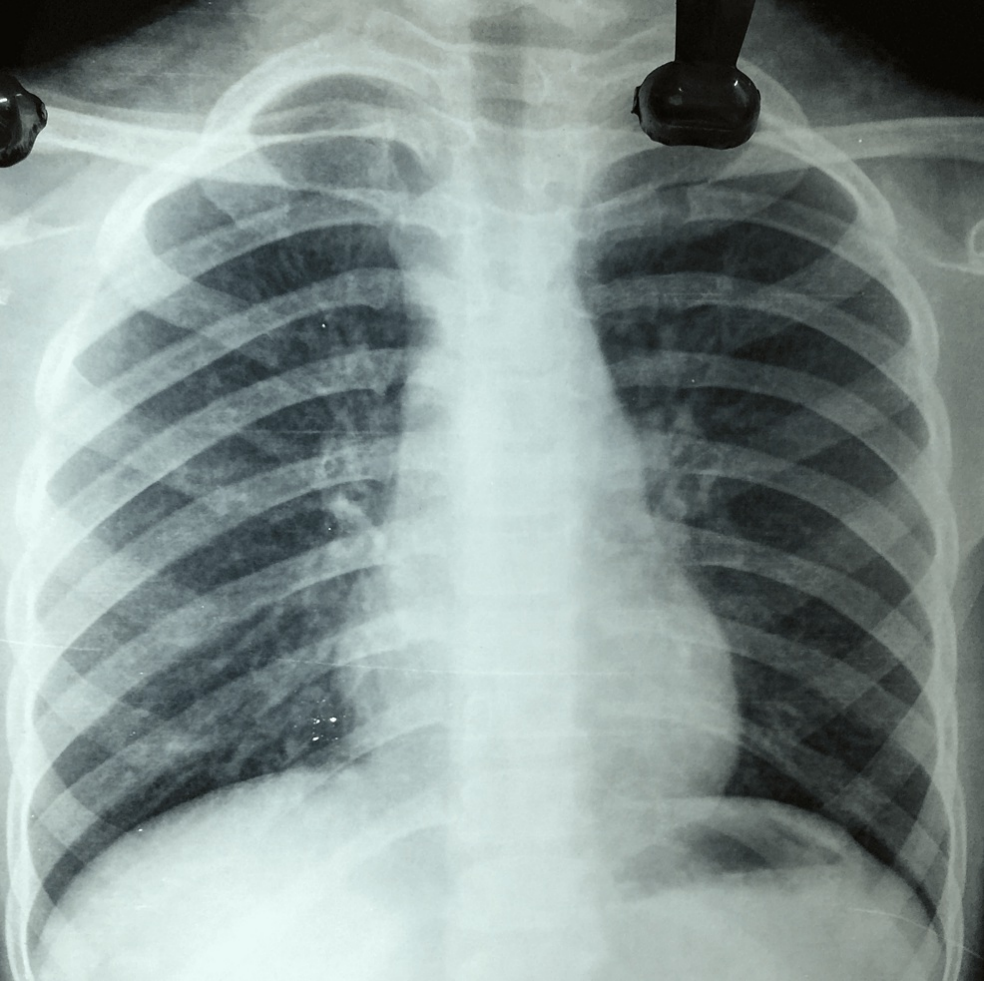
Chest radiograph PA view PA - posteroanterior

Laboratory results were within the normal limits for complete blood count, blood glucose, liver and kidney function, erythrocyte sedimentation rate, and urine test. HIV- I and II were non-reactive. Sputum for AFB was negative. A chest radiograph posteroanterior (PA) view was normal (Figure 2). The Mantoux test (15 x 15 mm) was strongly positive with induration of more than 10 mm.

Finally, based on the local examination of the lesion, family history, and biopsy findings, a diagnosis of lupus vulgaris was made. She was started on a fixed dose combination of antituberculous treatment as per her weight having a combination of isoniazid 300 mg, pyrazinamide 1,000 mg, rifampicin 450 mg, and ethambutol 600 mg, every morning for a period of eight weeks constituting the intensive phase followed by a 16-week continuation phase of isoniazid, rifampicin, and ethambutol. After treatment initiation, at her parent’s request, she was transferred out to her village.

## Discussion

Lupus vulgaris is an infrequently reported, chronic, progressive type of TB due to an incessant spread from an intrinsic focus or by hematogenous or lymphatic spread or rarely from exogenous sources (biologic liquids) such as infectious droplets [8]. Lupus vulgaris is predominantly reported in young adults on the face and neck [8]. The situation becomes difficult when the lesions are present in other body parts [8]. Facial involvement is commonly seen in western countries but in India, buttocks and extremities are commonly affected [5]. An asymptomatic scaly plaque developed by coalescing red-brown papules with a soft consistency termed lupoma is the characteristic of lupus vulgaris [8,10]. The plaque progresses gradually with the peripheral development of new papules [8]. A pale brownish-yellow or “apple-jelly” color on blanching by diascopic pressure is observed on the lesions [8]. And classic tubercles with or without caseation are classical findings on histological examination [8]. Epiluminescence microscopy, also known as dermatoscopy is an important diagnostic aid, where findings like finely focused telangiectasias on a yellow to the golden background are compared with the apple-jelly sign [11]. Further, the low sensitivity and specificity of PCR due to low bacillary load in lupus vulgaris limits its use [8]. In research laboratories, the amplification of different segments of the genome has been tested and it has been found that the insertion sequence IS6110 has a sensitivity that varies, depending on the study, between 70% and 90% and a specificity between 90% and 95%. An additional dot blot procedure increases the sensitivity and specificity of the PCR. However, it could be discrepant, assuming in the pandemic context of the case.

A case similar to this case was published by Theodosiou et al., where the diagnosis was established by dermatoscopic findings and skin biopsy [8]. The present case shares certain similarities like diagnosis by dermatoscopic findings and skin biopsy. However, the present case differs from their case in the location of the plaques, age, ethnicity, and also gender. One more case on the right wrist was reported by Mizuta et al., where an intrinsic focus due to pulmonary TB was noted [12]. This case differs from their case in the age, ethnicity, gender, and absence of pulmonary TB. Several other cases of lupus vulgaris are available in the literature but such presentations with appropriate management in a pandemic are rare.

The main aim of this case is to highlight the challenges faced during the third wave of COVID-19. It is widely reported that a number of other diseases were neglected during the pandemic. Dermatology outpatient departments were either shut down or were working with a very less workforce with e-consultation facilities (mostly beyond the reach of poor people) and the human resources were channeled toward COVID-19 care [13]. The health facilities and laboratory services were overwhelmed. Besides, the lockdowns and fear of COVID-19 forced the general population to stay indoors. Therefore, it became very difficult to get experts’ opinions and necessary lab investigations for a smooth diagnostic work-up in cutaneous TB cases. Additionally, if left untreated the lesions could progress to squamous cell and basal cell carcinomas or sarcomas [5]. Moreover, the complications of lupus vulgaris lesions include scarring, contractures, and tissue destruction or disfiguration [5].

## Conclusions

The author presented a case of lupus vulgaris in an Indian child. She was immunocompetent with no known major medical or surgical history. The main emphasis of this case is to stress the diagnosis and management of diseases that received little attention during COVID-19. The situation of a pandemic in its full bloom hampered prompt diagnosis and treatment of common infections like TB. The chaos due to mass-scale mortality and morbidity was remarkable and resulted in delayed presentations of certain diseases. Cutaneous TB if untreated could progress to carcinoma and thus a high degree of clinical suspicion is required to diagnose such cases, especially when there is delayed presentation and ambiguity in the test results or less common clinical symptoms, especially in the background of a pandemic.
